# The virtue of optimistic realism - expectation fulfillment predicts patient-rated global effectiveness of total hip arthroplasty

**DOI:** 10.1186/s12891-021-04040-y

**Published:** 2021-02-13

**Authors:** Anne Kästner, Virginie S. C. Ng Kuet Leong, Frank Petzke, Stefan Budde, Michael Przemeck, Martin Müller, Joachim Erlenwein

**Affiliations:** 1 Department of Anesthesiology, Pain Clinic, University Hospital, Georg August University of Goettingen, Robert-Koch-Str. 40, 37075 Göttingen, Germany; 2 Department for Orthopedic Surgery, Medical School, Hannover, Germany; 3 Department of Anesthesiology and Intensive Care, Annastift, Hannover, Germany

**Keywords:** Expectations, Hip replacement, Postoperative pain, Predictors of postoperative outcomes, Observational cohort study, Patient-reported outcomes

## Abstract

**Background:**

Emerging evidence highlights the importance of preoperative expectations in predicting patient-reported outcomes of orthopedic surgeries. To date, it is still a matter of controversy whether patient satisfaction can be maximized by promoting either optimistic or realistic outcome expectations before surgery. Adjusting overly optimistic outcome expectancies in favor of a more realistic outlook on the limitations of total hip arthroplasty could reduce the risk of disappointment and lead to greater satisfaction with surgery outcomes. Our prospective cohort study was aimed at comparing the relative predictive influence of baseline expectations, expectation fulfillment and symptomatic improvement on the global effectiveness of total hip arthroplasty.

**Methods:**

Ninety patients (49 female, 41 male; mean age: 63 ± 12.87 years) fulfilled inclusion criteria and completed a comprehensive preoperative assessment comprising sociodemographic, clinical, functional and psychological phenotypes. Moreover, the strengths of preoperative expectations for improvements in eight pain-related and functional domains were recorded on a 5-point Likert-scale. At 12 months after surgery, patients were asked to rate perceived improvements in each of these domains as well as the global effectiveness of the total hip replacement on a 5-point Likert-scale. To evaluate the relative impact of preoperative expectations, symptom improvement and the fulfillment of expectations on the global effectiveness of surgery, a sequential multiple regression analysis was performed.

**Results:**

Compared with the actual improvement at 12-months follow-up, prior expectations had been overly optimistic in about 28% of patients for hip pain, in about 45% for walking ability and around 60% for back pain, independence in everyday life, physical exercise, general function social interactions and mental well-being. An optimistic hip pain expectation, walking ability at baseline and the fulfillment of expectations for walking ability, general function and independence in everyday life were found to independently predict global effectiveness ratings.

**Conclusions:**

Positive expectation about pain and the fulfillment of expectations concerning functional domains predicted higher global effectiveness ratings. In line with many authors investigating the relationship between the fulfillment of expectations and satisfaction with medical interventions, we suggest that professionals should explicitly address their patients’ expectations during the preoperative education and consultation.

**Supplementary Information:**

The online version contains supplementary material available at 10.1186/s12891-021-04040-y.

## Background

Osteoarthritis is the most common degenerative joint disease among the elderly worldwide [1–3]. Owing to population ageing, prevalence rates are expected to continue rising generating considerable costs for the healthcare system [4]. Accordingly, utilization rates of total hip arthroplasty (THA) have been increasing over the last two to three decades in industrialized countries [5–7].

THA is indicated in patients suffering from end-stage osteoarthritis of the hip, inflammatory arthritis, fracture or dysplasia who do not respond to conservative therapies [5]. It is recognized as an effective surgical intervention for alleviating pain and improving mobility and quality of life in these patients [8, 9]. Yet, about one third suffer from persistent postoperative pain after THA and 3 to 16% report being dissatisfied with the outcome [10–14]. Thus, for quality management in competitive healthcare systems understanding and influencing modifiable determinants of patient satisfaction has become increasingly important [9]. Several preoperative risk factors for dissatisfaction with surgical outcomes have been identified with high consistency across studies: higher age, female gender, co-morbidities, associated conditions affecting walking capacity, mental distress, higher pain levels, and lower socioeconomic status [13, 15–18].

Patients’ expectations of treatment are increasingly acknowledged as an important determinant of the patient-rated effectiveness of the treatment outcome [19]. If not for the expectation of symptomatic improvement, few people would opt for having elective surgery. Scientific interest in preoperative expectations modulating patient-reported outcomes has been increasing over the last years producing largely inconsistent results [17]. Owing to the low methodological study quality and heterogeneity of construct definitions and measurements [17, 20–22], the exact nature of the relationship between expectations and outcome still remains a matter of controversy [20]. Some studies find patient satisfaction to be mainly predicted by postoperative improvement in symptoms and function, irrespective of prior expectations or expectation fulfillment [21]. Others state that high expectations per se favor better outcomes [23–25], possibly reflecting the influence of dispositional optimism and placebo effects [26–28]. Yet other findings emphasize the importance of the fulfillment of preoperative expectations, regardless of them being optimistic or pessimistic [21].

Evidently, it is crucial for surgeons to know whether to promote optimistic attitudes in their patients or whether to correct those in favor of a more realistic perspective on potential postoperative outcomes, given the individual constellation of risk factors present [20, 21]. Unfortunately, however, it is still not possible to derive consistent recommendations for the preoperative doctor-patient communication from the existing body of literature [20].

Consequently, there is a strong need for prospective investigations simultaneously addressing symptomatic improvement, expectations, and expectation fulfillment in multivariate models. Applying a sequential multiple regression model, Mannion et al. [21], among others [22, 29, 30] provided convincing evidence for the fulfillment of expectations as an independent predictor of the patient-rated global effectiveness of spinal surgery.

By translating the methodological approach of Mannion et al. to the field of total hip arthroplasty [21], this observational cohort study was aimed at examining the relative importance of three potential predictors of patient-rated treatment effectiveness: Preoperative outcome expectations (1), symptom relief/ functional improvement (2) and the fulfillment of outcome expectations (3). Moreover, we made sure to employ psychometrically sound instruments, widely applied and relevant to patients with hip osteoarthritis [9]. As suggested by Haanstra et al. in their review article [22], we additionally included psychological factors like catastrophizing and depression potentially confounding the association between expectations and outcome.

## Methods

### Participants

The study complies with the Declaration of Helsinki and the STROBE guidelines [31]. The Ethics Committee of the University Hospital Goettingen (No. 5 /4 /12) and the Ethics Committee of the Hannover Medical School (No. 1483–2012) approved the protocol. All patients gave written informed consent.

**Fig. 1:**
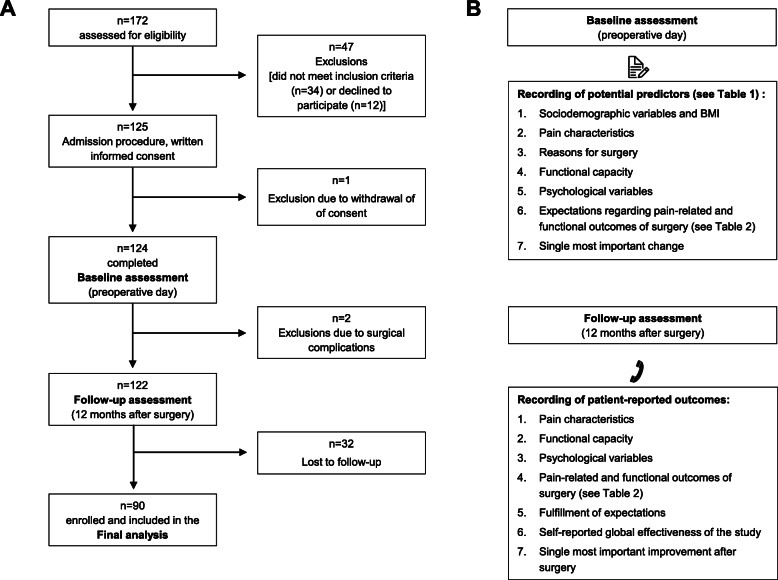
Study flow and overview of assessed variables **a**) Flow-chart, **b** Overview of the variables assessed at baseline and at 12 months follow-up

From July to November 2012, *N* = 172 consecutive patients scheduled to undergo total hip arthroplasty at the Orthopedic Clinic of the Hannover Medical School were screened for eligibility. Inclusion criteria were: (a) age ≥ 19, (b) fluent German literacy skills, (c) the mental and legal ability to give written consent and (d) the agreement to participate in the 12-month follow-up survey and to provide contact details. Patients suffering from dementia, planned spinal anesthesia (all included patients received balanced general anesthesia), drug addiction and post-surgical delirium syndrome were not eligible. We further excluded patients suffering from peri- and postoperative complications (such as post-operative delirium, and prosthetic joint infection) from the 12-month follow-up analysis (for an overview of the study protocol see Fig. 1). In total, *N* = 90 patients were included for the statistical analysis, *n* = 82 had to be excluded or were lost to follow-up (see Fig. 1 for further information). The baseline characteristics of the study participants are shown in Table 1.

**Table 1:** Sociodemographic, pain-related, functional and psychological variables at baseline

Sociodemographic variables and Body Mass Index	
Gender, *No.*	49 women; 41 men
Age at examination, years, *mean ± SD*	63 ± 12.87
Degree of school education, *No. (%)*	
No graduation	1 (1%)
„Haupt−/Volksschule “^*a*^	36 (40%)
„Realschule/mittlere Reife“^b^	27 (30%)
„Fachhochschule, Abitur, allg. Hochschule“^c^	26 (29%)
Body Mass Index (BMI)*,* kg/m^2^; *mean ± SD*	27.57 ± 4.66
**Pain characteristics**	
Average hip pain in the last 3 months before surgery, Numeric Rating Scale (0–10), *median (1st; 3rd quartile)*	6 (4; 7)
Overall severity of chronic pain condition, Chronic Pain Grade (von Korff), *No. (%)*	
Grade 1	15 (17%)
Grade 2	20 (23%)
Grade 3	17 (18%)
Grade 4	38 (42%)
Pain chronicity, MPSS^d^, No. *(%)*	
Stage I (low)	36 (40%)
Stage II (medium)	37 (41%)
Stage III (high)	17 (19%)
Duration of hip pain, time intervals, No. *(%)*	
1 to 12 months	18 (20.1%)
12 to 24 months	22 (24.4%)
2 to 5 years	31 (34.4%)
More than 5 years	19 (21.1%)
Pressure pain threshold (PPT), kPa, *mean ± SD*	391.55 ± 179.35
**Functional capacity**	
Walking ability, Timed up and go test score, *median level, (1st; 3rd quartile)*	2 (2; 2)
Hip function and mobility, WOMAC^e^ score*, mean ± SD*	53.09 ± 20.82
**Psychological variables**	
Health-related quality of life, SF-12^f^, *mean ± SD*	
SF-12 Physical	29.89 ± 7.85
SF-12 Mental	49.23 ± 12.35
Psychological distress, DASS^g^, *median (1st; 3rd quartile)*	
DASS Depression	3 (1; 5)
DASS Anxiety	1 (0; 3)
DASS Stress	5 (2; 8)
Somatization, PHQ-15^h^, *median (1st; 3rd quartile)*	5 (4; 8)
Kinesiophobia, TSK^i^, *median (1st; 3rd quartile)*	36 (31; 41)
Cognitive appraisal of pain*,* KPI^j^, *median (1st; 3rd quartile)*	
Catastrophizing thought scale	2 (0.47; 3.11)
Helplessness scale	0.4 (0; 1.20)
Thought suppression scale	2.75 (1.25; 3.75)
Fear of surgery, Numeric Rating Scale (0–10), *median (1st; 3rd quartile)*	3 (1; 6)
Fear of pain after surgery, Numeric Rating Scale (0–10), *median (1st; 3rd quartile)*	2 (1; 5)

Descriptive statistics are based on *N* = 76–90 subjects due to varying numbers of missing data per variable

^a^”Hauptschule” in Germany refers to the final examination at grade 9

^b”^Realschule” finishes after grade 10 with the degree “mittlere Reife”

^c”^Gymnasium” finishes with the final examination called “Abitur” after grade 13

^d^*MPSS* Mainz Pain Staging System (Schmitt et al., [32])

^e^*WOMAC* Western Ontario and McMaster Universities Osteoarthritis Index (Stucki et al., [33])

^f^*SF-12* short form of the Health Survey Questionnaire (Jenkinson et al., [34])

^g^*DASS* Depression, Anxiety, Stress Scales (Nilges und Essau, [35])

^h^*PHQ-15* Patient Health Questionnaire (Kroenke et al. [36])

^i^*TSK* Tampa Scale for Kinesiophobia (Roelofs et al. [37])

^j^*KPI* Kiel Pain Inventory (Hasenbring, [38])

### Preoperative procedure (assessment at baseline)

On the preoperative day, patients were interviewed and physically examined by the study physicians with the aim of recording basic sociodemographic and clinical data. Tests of hip mobility and function and the pressure pain threshold were conducted. A comprehensive questionnaire booklet was administered at baseline addressing potentially relevant predictors of patient-reported global effectiveness of THA. In addition to the phenotypes detailed in the left-hand column of Table 1, the booklet included questions concerning outcome expectations (see Table 2) and the single most important change for the surgery to be judged as successful by the patients.

**Table 2:** Comparison of patients’ expectations at baseline and respective outcomes 12 months after total hip arthroplasty (THA)

	Percentage of patients (*N* = 90) in each category at baseline (*pre*) and 12 months (*12 m*) after THA											
	Much better		Better		Somewhat better		Unchanged		Worse		Uncertain	
	*pre*	*12 m*	*pre*	*12 m*	*pre*	*12 m*	*pre*	*12 m*	*pre*	*12 m*	*pre*	*12 m*
Hip pain	79	66	21	30	–	2	–	2	–	–	–	–
Back pain	37	6	34	30	**7**	**30**	**9**	**33**	–	1	13	–
Walking ability	**83**	**48**	**15**	**36**	1	14	–	1	–	1	1	–
Independence	**67**	**33**	**25**	**46**	2	19	2	2	–	–	4	–
Physical exercise	**44**	**15**	35	28	9	27	7	30	–	–	5	–
General function	**59**	**21**	37	42	**1**	**29**	3	8	–	–	–	–
Social interactions	**32**	**2**	30	20	**5**	**40**	27	38	–	–	6	–
Mental well-being	**39**	**5**	36	31	**2**	**40**	21	23	–	1	2	–

Discrepancies between patients‘expectations at baseline and respective outcomes at 12 months after THA that exceed 20% are marked in bold

#### Expectations regarding pain-related and functional outcomes of the surgery

Expectations of the surgery outcomes were evaluated at baseline using a modified version of the validated and psychometrically sound “Expectation Scale” from the North American Spine Society (NASS) Lumbar Spine Questionnaire [39]. Patients were asked to report their expectations regarding the following pain-related and functional surgery outcomes on a 6-point scale (I don’t know; worse = 1; unchanged = 2; somewhat better = 3; better = 4; much better = 5): Hip pain, back pain, walking capacity, independence, physical exercise, everyday functioning, social interaction and mental well-being.

#### Single most important outcome

The single most important individual outcome occurring after the surgery in order for patients to judge the THA as successful was also recorded before surgery. Answer possibilities were: Improvements in hip pain, back pain, walking capacity, independence, physical exercise, everyday functioning, social interaction and mental well-being [21] .

#### Reasons for surgery

Patients were asked to choose their 3 most important reasons for undergoing THA from the following options: Other therapies were ineffective, something must be done, fear of worsening of my situation, to retain my independence, to improve everyday functioning, to improve walking capacity, to reduce pain, my physician recommended the surgery.

#### Pain characteristics

The average hip pain intensity in the last 3 months before surgery was assessed on an 11-point Numeric Rating Scale (NRS; 0 = no pain to 10 = worst pain imaginable) [40]. The severity of chronic pain was operationalized by use of the Chronic Pain Grade (CPG) which models the relationship between pain intensity and disability [41]. The CPG grades pain severity into four hierarchical categories: Grade 1: Low disability and low pain intensity; Grade 2: Low disability and high pain intensity; Grade 3: High disability, moderately limiting and Grade 4: High disability, severely limiting.

Pain chronicity stages (I-III, acute to chronic pain) were derived using the validated Mainz Pain Staging System (MPSS) [42, 43]. The classification is based on a 10-item-questionnaire rated by the study physicians. It takes into account temporal and spatial dimensions of pain (over a 4-week recall period), the history of medication usage and the life-time utilization of the health care system [42].

As a measure of overall pain sensitivity, the pressure pain threshold (PPT) was recorded using an electronic pressure algometer (Somedic Production, Stockholm, Sweden) bilaterally over five sites (thumb, lateral epicondylus, upper division of the trapezius, quadriceps femoris, and tibialis anterior) [44, 45]. The algometers’ probe tip (1 cm^2^) was applied to each site. Patients were advised to indicate when first perceiving pain during pressure stimulation with slowly increasing intensity (50 kPa/s). Pressure stimulation stopped at the patients’ report of pain or when maximum pressure intensity (1000 kPa) was reached. Analyses are based on the average threshold (kPa) over all 10 testing sites.

#### Functional capacity

In order to measure the patients’ individual mobilization ability, the psychometrically well evaluated Timed up and Go test was employed [46]. The time it takes for a person to stand up from a sitting position, walk three meters, turn around, walk back to the chair and sit down again is recorded. According to the time taken to complete the task, patients were assigned to 5 levels of mobility: Level 1: independent mobility (< 10 s); Level 2: mostly independent mobility (< 20 s); Level 3: variable mobility (20–29 s); Level 4: impaired mobility (> 30 s); Level 5: unable to walk or to fulfill the task.

To evaluate the functional capacity of the patients, the German version of the „Western Ontario and McMaster Universities Osteoarthritis Index” (WOMAC) was employed [33]. The WOMAC is a reliable and valid, self-administered instrument assessing the items pain, stiffness and physical functioning of patients suffering from knee or hip osteoarthritis. Higher scores representing more pronounced functional disability.

#### Psychological variables

Health-related quality of life was assessed using the German version of the Short-Form Health Survey (SF-12) including physical (Physical Component Summary Score - PCS) and mental health (Mental Health Component Summary Score - MCS) [34, 47]. Summary scores range between 0 and 100 for both PCS and MCS [48]. A value of about 50 represents the mean of a standard population, higher values represent better health-related quality of life [34], a value difference of 10 represents a standard deviation.

Psychological distress in terms of depression, anxiety and stress was measured employing the German version of the Depression, Anxiety, Stress Scales (DASS) [35, 49, 50]. The DASS has acceptable psychometric properties and is made up of three subscales, each comprising 7 items to be rated on a 4-point Likert-type scale with higher values indicating higher psychological distress.

Somatization has previously been associated with substantial functional impairment and healthcare utilization [51, 52]. In our study, somatization was evaluated by means of the German version of the Patient Health Questionnaire (PHQ) [36]. The PHQ-15 inquires about the severity of 15 somatic symptom clusters which include 14 of the 15 most prevalent DSM-IV somatization disorder somatic symptoms on a 3-point scale (0 = not bothered at all, 1 = bothered a little and 2 = bothered a lot). The PHQ-15 total score represents the sum of the individual items ranging from 0 to 30.

Movement-related fear (“kinesiophobia”) has been increasingly recognized as a crucial predictor of the maintenance of pain and disability [53, 54]. We used the German version of the “Tampa Scale for Kinesiophobia” (TSK) which has good psychometric properties [37, 55]. It contains 17 questions rated on a 4-point Likert-type scale (total score range 17 to 68). The fear of surgery and of postoperative pain was additionally measured on an 11-point NRS (0 = no fear and 10 = worst fear imaginable).

The cognitive appraisal of pain was assessed using the Kiel Pain Inventory (KPI) [38]. It contains three independent self-rating instruments for the standardized assessment of the cognitive, emotional and behavioral processing of pain: The Catastrophizing Thought Scale (CTS; 5 items), the Thoughts of Helplessness Scale (THS; 9 items) and the Thought Suppression Scale (TSS; 4 items). Items are rated on a 7-point Likert-type scale and the subscale scores represent the mean of the respective items.

### Postoperative procedure (assessment at 12-months follow-up)

At 12 months after surgery, average hip pain intensity in the last 3 months (NRS), overall severity of chronic pain (CPG), functional capacity (WOMAC) and psychological outcome variables (health-related quality of life, psychological distress and kinesiophobia) were recorded again using the respective standardized questionnaires (SF-12, DASS and TSK) in a telephone interview. In addition, the following outcome parameters were assessed according to a standardized protocol:

#### Self-reported global effectiveness of the surgery

At follow-up, patients rated the global effectiveness of the surgery (“How did the surgery help you overall?”) on a 5-point scale (made it worse, did not help, helped a bit, helped, and helped a lot).

#### Single most important positive outcome after surgery

Patients were additionally asked to state the single most important positive outcome they experienced as a result of the THA (response categories: Improvements in hip pain, back pain, walking capacity, independence, physical exercise, everyday functioning, social interaction and mental well-being) [21].

#### Pain-related and functional outcomes of surgery (see Table 2)

Patients were asked to rate the actual improvements regarding the 8 outcome parameters asked for in the preoperative examination booklet (expectations regarding pain-related and functional outcomes of surgery) on a 5-point scale (1 = worse, 2 = same, 3 = somewhat better, 4 = better and 5 = much better).

#### Fulfillment of expectations

**Fig. 2:**
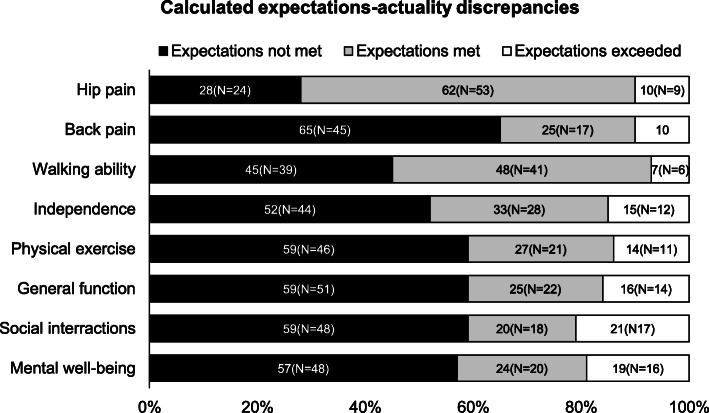
Calculated expectations-actuality discrepancies. Expectations-actuality discrepancies were obtained by subtracting the preoperative expectations for the eight outcome parameters from their actual improvement at 12-month follow-up. The resulting scores (range: − 5 to 5) were divided into three groups (score < 0: expectations not met, score = 0: expectations met and score > 0: expectations exceeded). The graph shows the percentages of patients for whom expectations were not met, met or exceeded

The fulfillment of expectations was obtained by subtracting the preoperative expectation score for each outcome parameter from the respective postoperative outcome score. The resulting expectations-actuality discrepancy scores ranged from − 5 to 5 points. A negative expectations-actuality discrepancy score indicated less improvement than expected and was termed “expectations not met”. A score of zero indicates an outcome as expected and was termed “expectations met”. A positive expectations-actuality discrepancy represents a greater improvement than expected and was named “expectations exceeded” (Fig. 2).

### Statistical analyses

Statistical analyses were carried out using SPSS software (IBM SPSS Statistics for Windows, version 21.0; IBM Corp, Armonk, NY). All reported *p*-values are two-sided and the level of significance was set to *p* < 0.05. Continuous variables were described by mean and standard deviation. Discrete variables were presented as frequencies (nominal data) or median with first and third quartiles (ordinal data). The distribution of continuous data was tested for normality using the Kolmogorov-Smirnov-Test.

Symptom change scores for pain characteristics, functional capacity and psychological variables were obtained by dividing the outcome measures at 12-months follow-up by the preoperative scores. Associations of baseline characteristics, preoperative expectations, symptom change scores and the fulfillment of expectations with the patient-rated global effectiveness of surgery, were explored by correlation analyses (Supplementary Tables 1 and 2). Multiple testing was accounted for by Bonferroni correction. Cohen’s Kappa (κ) was used to determine the agreement between identical rating (e.g. preoperative expectations and postoperative change). For metric data, Pearson’s r and for dichotomous variables (i.e. gender) phi correlation coefficient (Chi-squared test of Pearson) was used. Ordinal data were correlated using the non-parametric rank test Kendall Tau-B (τB) as it has been shown to be more robust and slightly more efficient than Spearman rank-order correlation coefficient [56, 57].

To evaluate the relative predictive influence of preoperative expectations, symptom change scores and the fulfillment of expectations on the patient-rated global effectiveness of surgery (dependent variable) a multiple regression analysis was performed adjusting for multiple baseline characteristics, performing both a complete case analysis (Supplementary Table 3) and an analysis with imputation of missing data (Table 3). Displayed regression coefficients were mutually adjusted for the respective other predictors. Variables significantly correlated with the dependent variable after Bonferroni-adjustment for multiple testing in prior correlation analyses (see Supplementary Tables 1 and 2) were entered into the multiple regression model in a stepwise approach. The gain in explained variance (R^2^) from one step to the next is reported.

**Table 3:** Results of the sequential multiple regression analysis (*N* = 85^a^): Variance explained in the global effectiveness of total hip arthroplasty at 12 months follow-up by sociodemographic and medical variables, preoperative expectations, change in symptoms and the fulfilment of expectations (multiple imputation of missing values)

	Variables included upon each step	Range of R^**2**^ across the 10 imputed data sets	Pooled β (p-value) in final model (only significant predictor variables shown)
**Step 1** Confounding factors	Degree of school education Average hip pain in the last 3 months before surgery Overall severity of chronic pain (CPG) Hip function and mobility (WOMAC) Walking ability (Timed up and go score)	0.261–0.304	–
**Step 2** Preoperative expectations	Degree of school education Average hip pain in the last 3 months before surgery Overall severity of chronic pain (CPG) Hip function and mobility (WOMAC) Walking ability (Timed up and go score) **Preoperative hip pain expectation**	0.372–0.416	β = −2.143 (*p* = 0.033) **Walking ability (Timed up and go score)** β = 3.745 (*p* < 0.001) **Preoperative hip pain expectation**
**Step 3** Improvement in symptoms (Symptom change scores)	Degree of school education Average hip pain in the last 3 months before surgery Overall severity of chronic pain (CPG) Hip function and mobility (WOMAC) Walking ability (Timed up and go score) Preoperative hip pain expectation **Symptom change scores:** **Average hip pain in the last 3 months** **hip function and mobility (WOMAC)** **Health-related quality of life (SF-12 physical)**	0.419–0.493	β = − 2.338 (*p* = 0.020) **Walking ability (Timed up and go score)** β = 3.370 (*p* = 0.001) **Preoperative hip pain expectation**
**Step 4** Fulfillment of expectations (calculated expectations-actuality discrepancy scores)	Degree of school education Average hip pain in the last 3 months before surgery Overall severity of chronic pain (CPG) Hip function and mobility (WOMAC) Walking ability (Timed up and go score) Preoperative hip pain expectation Symptom change scores: Average hip pain in the last 3 months Hip function and mobility (WOMAC) Health-related quality of life (SF-12 physical) **Calculated expectations-actuality discrepancy scores:** **Walking ability** **Independence** **Physical exercise** **General function** **Social interactions**	0.613–0.689	β = −3.103 (p = 0.002) **Walking ability (Timed up and go score)** β = 4.605 (*p* < 0.0001) **Preoperative hip pain expectation** β = 2.601 (*p* = 0.009) Fulfillment of expectations (calculated expectations-actuality discrepancy scores): **Walking ability** β = 2.952 (*p* = 0.002) Fulfillment of expectations (calculated expectations-actuality discrepancy scores):: **Independence** β = − 2.783 (p = 0.006) Fulfillment of expectations (calculated expectations-actuality discrepancy scores): **General function**
**Final model** (only significant predictors included)	Walking ability (Timed up and go score) Preoperative hip pain expectation Calculated expectations-actuality discrepancy scores: Walking ability Independence General function	0.510–0.544	

^a^Individuals with more than 3 out of 14 missing predictors were excluded from the analysis. The variables marked in bold are add up to the model at each step. Predictor variables individually significantly associated with global effectiveness of THA (see Supplementary Table 1 and 2) were entered in four steps. The significant predictors in the final model were: Hip pain expectation, walking ability at baseline and the calculated expectations-actuality discrepancy scores of walking ability, general function and independence in everyday life

β in final model = β regression coefficient after all listed variables have been entered; R2change = Increase in explained variance by step. Level of significance was set to *p* < 0.05

#### Multiple regression model and imputation procedure

In a first step of the multiple regression, potentially confounding baseline characteristics were entered. In a second step, items from the expectations scale significantly associated with the primary outcome measure in the prior univariate analysis were added. In a third step, symptom change scores (quotient of outcome measures at 12-months follow-up and preoperative scores of the respective questionnaires) were entered into the model. In a fourth step, expectations-actuality scores were added.

Due to varying numbers of missing data for the different predictor variables, the sample size decreased with increasing number of variables included. As this may reduce statistical power and lead to biased results, we replicated the sequential multiple regression analysis following multiple imputation of missing values (Table 3). In preparation of the multiple imputation, the patterns of missing values were analyzed (Supplementary Fig. 1) and judged to be randomly distributed. Multiple imputations were carried out for individuals with missing values for less than three out of 14 predictors (*N* = 85).

Ordinal and categorical variables were imputed based on logistic regression models, continuous variables were imputed based on linear regression models using the automatic imputation algorithm of the SPSS software package (IBM SPSS Statistics for Windows, version 21.0; IBM Corp, Armonk, NY) which is based on the MAR (missing at random) assumption. As White et al. [58] suggest that the number of imputations should be larger than the percentage of missing values (in our study approx. 6%), 10 imputations were used. For each step of the sequential analysis, the range of explained variance (R^2^) across the 10 imputed complete data sets and the pooled beta regression coefficients for the statistically significant predictors are presented (Table 3).

The missing values analysis revealed that for 93% of the predictors included at step 4 of the regression model at least 1 value is missing. Moreover, 40% (*N* = 36) of the study participants had at least one missing value on a variable, so that 6% of the 1260 values (cases × variables) were missing and had to be imputed. The results from the complete case analysis could be replicated in the 10 imputed data sets with comparably high goodness-of-fit according to Cohen [59].

## Results

### Description of the study sample

Data analyses were based on 90 patients admitted to the hospital for total hip replacement surgery (right side: *N* = 46; left side: *N* = 44). The most frequent indication for surgery was primary osteoarthritis of the hip (77%, *N* = 67), followed by hip dysplasia (15%, *N* = 13), femoral head necrosis (5%, *N* = 4) and posttraumatic osteoarthritis (3%, *N* = 3). On average, patients reported moderate pain intensities. Only one third of patients were classified as patients with a high level of chronicity. The majority of the cohort still had independent mobility skills. Symptom load for psychological parameters such as depression, somatization, kinesiophobia, catastrophizing, helplessness and fear of surgery or pain was rather low (below the cut-offs of clinical relevance). For a comprehensive sociodemographic and clinical characterization, see Table 1.

### Reasons for surgery

The most common reason for deciding to undergo surgery was “to improve walking capacities” (25%, *N* = 63), followed by “to reduce pain” (21%, *N* = 53), “to retain my independence” (18%, *N* = 47), to “improve everyday functioning” (15%, *N* = 39), “other treatments were ineffective” (9%, *N* = 24), “fear of worsening of my situation” (8%, *N* = 20) and “my physician recommended it” (4%, *N* = 10).

### Importance of the expected positive outcomes

To evaluate the surgery as a success, 47% (*N* = 39) of the participants found improvement in hip pain to be the most important expected positive outcome (walking ability: 30%(*N* = 25), general function: 14%(*N* = 12), independence in everyday life: 7% (*N* = 6), back pain: 1% (*N* = 1) and physical exercise: 1% (*N* = 1). This preoperative rating correlated significantly (*p* = 0.006, κ = 0.188) with the patients’ follow-up rating of the most important positive outcome that had occurred after the surgery (hip pain: 59% (*N* = 53), walking ability: 24% (*N* = 21), general function: 7% (*N* = 6), independence in everyday life: 6% (*N* = 5), back pain: 3% (*N* = 3) and physical exercise: 1% (*N* = 1).

### Preoperative expectations and postoperative outcomes

The domain-specific preoperative expectations and respective postoperative outcomes after 12 months are shown in Table 2. Consistent with the most important reasons for undergoing surgery, expectations for improvements in hip pain, physical and general functioning and independence in in everyday life were rather high. With respect to back pain, social functioning and mental well-being, expectations were slightly less optimistic. Accordingly, at 12-months follow-up, most of the patients reported an improvement (“much better”, “better” or “somewhat better”) in hip pain and walking ability and independence in everyday life. The largest discrepancy between baseline expectations and postoperative outcome was reported for back pain and physical exercise. Social interactions showed an improvement in 62% (*N* = 56) of the participants.

### Fulfillment of expectations

While for hip pain, 62% (*N* = 53) of patients reported having met their expectations, in 10% (*N* = 9) actual improvement exceeded their preoperative expectations. Similarly, 48% (*N* = 41) of patients reported meeting their expectations concerning walking ability while 7% (*N* = 6) exceeded their expectations. More than 50% of patients were overly optimistic in their expectations of actual improvement concerning back pain (*N* = 45), independence in everyday life (*N* = 44), physical exercise (*N* = 46), general function (*N* = 51), social interactions (*N* = 48) and mental well-being (*N* = 48) 12-months after surgery (Fig. 2).

### Patient-rated global effectiveness of surgery at 12 months follow-up

When asked for the degree to which the total hip arthroplasty helped overall, responses were as follows: Helped a lot: 60% (*N* = 54), helped: 38% (*N* = 34), helped somewhat: 1% (*N* = 1), did not help: 1% (*N* = 1). No one thought that the surgery “made it worse”.

### Predictors of the patient-rated global effectiveness of surgery

To evaluate the relative predictive influence of preoperative expectations, symptom change and the fulfillment of expectations on the global effectiveness of surgery (dependent variable), a sequential multiple regression was performed. The multiple regression analysis was carried out based on the multiple imputation of missing data (Table 3; for complete case analysis, see supplementary Table 3) to account for the high rate missing values leading to a substantial loss in power and limiting the external validity of the predictor selection [60]. Baseline variables found to significantly correlate with the dependent variable (Supplementary Table 1) were included as confounders in the first step of the model. At step 1, no significant predictor was found (Table 3). As a next step, preoperative expectations significantly associated with the dependent variable were added (only hip pain expectation, Supplementary Table 2). At step 2, walking ability at baseline (timed up and go score) and preoperative hip pain expectation were found to significantly contribute to the model. Then, changes in symptoms (quotient of outcome measures at 12 months follow-up and preoperative scores of the same variable) significantly associated with the outcome was added (Supplementary Table 2). None of the symptom change scores resulted to be independent predictors of the patient-rated global effectiveness. As a last step, the domain-specific expectation fulfillment scores (calculated expectations-actuality discrepancy) for walking ability, independence, physical exercise, general function, and social interactions were entered. These lead to the highest change in explained variance. Importantly, in addition to the hip pain expectation and walking ability at baseline, expectation fulfillment in walking ability, independence in everyday life and general function appeared as independent predictors of the patient-rated global effectiveness of THA. The final model, including these five significant predictors explained about 52% of the variance in the patient-rated global effectiveness of surgery (Table 3).

## Discussion

The present study was designed with the aim to delineate to which extent patient self-ratings of treatment effectiveness were explained by preoperative outcome expectations, the fulfillment of these expectations or the improvement in core symptoms of hip osteoarthritis. Our analysis shows that patients undergoing total hip replacement surgery base their global effectiveness ratings mostly on the fulfillment of their preoperative outcome expectations. Interestingly, in our study, this was only observed for functional parameters like walking ability, general function and independence in everyday life. Additionally, we found the preoperative expectation of hip pain and walking ability at baseline to significantly predict the patient-rated global effectiveness of surgery. The improvement in core symptoms of hip osteoarthritis was not found to be an independent predictor of the global effectiveness of THA, however.

Our findings concur with a recent review on the role of expectation fulfillment in knee and hip arthroplasty [61]. It concludes that in only half of the studies preoperative expectations were associated with the level of satisfaction, while in 93% of the studies the fulfillment of expectations was shown to independently predict satisfaction with a hip or knee replacement surgery [61].

Interestingly, while for more complex functional constructs like walking ability, general function and independence in everyday life, the fulfillment of expectations was found to be crucial, for hip pain, an optimistic expectations per se appeared to be most beneficial for evaluating the surgery as a success - independent of its fulfillment. This finding gives reason to assume that expectancy effects work differently for non-volitional, sensory-affective experiences like pain and more complex functions. Experimental research on placebo effects has contributed convincing evidence for non-volitional responses being mostly modulated by the expectancy of their occurrence [62]. Thus, positive expectations about pain and pain-mitigating treatments have the potential to attenuate the subjective experience of pain by influencing attentional processes as well as affective appraisals leading to an activation of descending control systems [63, 64]. Expectations of functional recovery, such as walking ability and independence, are part of a much broader and abstract concept, which pertains more strongly to self-efficacy expectations and motivational mechanisms [62, 65, 66]. Functional recovery after THA depends on a multitude of factors presumably less subject to non-volitional processes [66]. It may rather be influenced by the surgical intervention itself increasing the mobility of the hip joint, the quality of the postoperative rehabilitation and, most importantly, motivational factors and self-efficacy expectations of the patients [62, 65].

### Limitations

Despite many salient features like the prospective nature, the use of standardized outcome measures and the comprehensive clinical characterization of the sample, the results of the present study have to be interpreted in the light of some limitations.

First of all, the varying numbers of missing data for the different predictors included in the sequential multiple regression analysis has to be mentioned as a key limitation of our study. Upon stepwise addition of increasing amounts of potential predictors, the number of observations decreased which limits the external validity of the predictor selection by potentially introducing biases and power issues. The model at step 4 (containing 14 independent variables) relying on complete case analysis was only based on 54 individuals and might have been underpowered for detecting small effects. Therefore, we provided a sensitivity analysis essentially replicating the results of the complete case multiple regression analysis following multiple imputation of missing values. Importantly, however, to run models with 14 predictors on *N* = 85 individuals does not comply with the generally accepted recommendation to ensure sufficient statistical power (the number of observations should be 10 times the number of independent variables) [22].

A central limitation in our and likely other investigations concerns more general aspects of the assessment of preoperative outcome expectations, mostly independent from the actual type of orthopedic surgery. Between 2012 and 2020 several review articles summarizing the available evidence on the predictive influence of preoperative expectations on patient satisfaction with THA have been published [17, 20, 22, 67]. All of them emphasize the need for a theoretical framing of the construct “patient expectations” and a consistent use of valid measurements. Several definitions of “expectations” relevant to the context of healthcare have been derived from theoretical developments in marketing psychology (“consumer satisfaction”) and biomedical research on placebo effects [68, 69]. Thompson and Suñol [69], for example, differentiate between predicted, ideal and normative expectations. A similar framework was proposed by Kravitz [70]. In the context of orthopedic surgery, “predicted expectations” can be defined as a patient’s likelihood estimation of symptom relief based on the information provided by the physician in the shared decision-making process before surgery [68, 69]. “Ideal expectations” reflect the patients’ wishes and desires while partly neglecting the odds of a good outcome. “Normative expectations” are defined as socially endorsed evaluations of what should be received from health services [23, 68]. Obviously, dependent on the exact wording of the question for measuring outcome expectations, different dimensions of the multifaceted construct can be preferentially targeted [69]. By employing the question “What changes in the following items do you expect to experience as a result of the surgery?” it is not clear which aspect of expectations (predicted, ideal or normative) we addressed in our study which limits construct validity and complicates the interpretation of results.

Moreover, we adapted the expectation scale of the NASS Lumbar Spine Questionnaire (which is validated for spinal surgery) for patients undergoing hip arthroplasty [17]. The adaption without validation limits the validity of our results. Nevertheless, all items assed (hip pain, back pain, walking ability, independence, physical exercise, everyday functioning, social interaction and mental well-being) are relevant and central factors concerning postoperative recovery after THA. Alternatively, we could have employed the validated Hospital for Special Surgery (HSS) Total Hip Replacement Expectations Survey comprising 18 items informed by the ICF-framework [71, 72]. However, the German, culturally adapted version of this questionnaire had not been validated until 2016 and was not available at the time of the planning and data collection of our study [73]. Future investigations should make use of the existing validated surveys of expectations and define which aspect of the construct they would like to address in order to obtain more reliable, comparable and accurate results.

Likewise, for the assessment of the patient-rated global effectiveness, we used a question that had not been previously validated which limits the internal validity of our results.

Additionally, some methodological issues have to be acknowledged. We lost 8% of the study cohort to follow-up for unknown reasons. Compared to other prospective cohort studies, the drop-out rate in our case was rather low [74]. Still, we cannot exclude the risk of attrition bias due to a selective drop-out of non-responders which would lead to a slight overestimation of outcome evaluations and global effectiveness ratings.

### Clinical implications

The improvement of patient satisfaction with surgery outcomes has important economic implications. Thus, paying more attention to the refinement of the preoperative patient education is indispensable [30, 75]. In the context of hip replacement surgeries or other medical interventions, large inter-individual variability can be found as a function of many factors such as progression of the medical condition, comorbidities and sociodemographic status [17]. In view of these unresolved uncertainties, how to go about communicating with the patients before surgery? Especially for those medical services people do not have prior experience with, expectations are often unformed and represented on a subconscious level [69]. Consequently, surgeons should explicitly inquire about expectations for different areas of daily functioning relevant to quality of life. Moreover, as can be derived from our findings and the literature [61], overly optimistic expectations with respect to hip pain do not have to be dampened as they resulted as an independent predictor of the patient-rated global effectiveness of surgery. Given the large expectations-actuality discrepancies for back pain, physical exercise and associated functions, we strongly suggest adjusting unrealistic expectations. At the same time, self-efficacy expectancies should be instigated in patients by teaching multimodal, self-effective strategies to promote functional recovery and by highlighting the importance of complying with the physical therapy regimen [65, 76].

## Conclusion

While the intricacies of the underlying mechanisms still need further research, expectations and the fulfillment of expectations are clearly pivotal factors in predicting the effectiveness of an intervention in a clinical setting. It is therefore critical for medical professionals to not only give detailed information about the process, results and risks of a medical intervention, but also to explicitly address expectations. Our data provide evidence for the beneficial effect of promoting an attitude of realistic optimism during the shared decision-making process before surgery.

## Supplementary Information

**Additional file 1: Supplementary Figure 1.** Missing value analysis of the primary outcome measure and the 14 predictor variables included sequentially into the multiple regression analysis. A. The table displays the descriptives (mean and standard deviation), the percentage of missing values per variable, the number of imputed values (missing values x number of imputations) and the type of model used for multiple imputation. For the dependent variable “global effectiveness of total hip arthroplasty” the data set was complete. More than 10% missing values were identified for the rating instrument measuring hip function and mobility (WOMAC), its symptom change score (quotient of postoperative and preoperative WOMAC score) and the calculated expectations-actuality discrepancy scores (fulfillment of expectations) of physical exercise and social interactions. B. The variable chart shows that for 93% of the predictors included at step 4 of the regression model at least 1 value is missing. The cases chart shows that 40% (*N* = 36) of the study participants has at least one missing value on a variable. The values chart shows that 6% of the 1260 values (cases × variables) are missing. C. The bar graph displays the percentage of study participants having none to six missing predictors. Subjects with more than three missing predictors were excluded from the analysis on the imputed data set.

**Additional file 2: Supplementary Table 1.** Correlations between baseline variables and patient-rated global effectiveness of THA.

**Additional file 3: Supplementary Table 2.** Correlations between preoperative expectations, change in symptoms and calculated expectations-actuality discrepancy scores with the patient-rated global effectiveness of THA.

**Additional file 4: Supplementary Table 3.** Results of the sequential multiple regression analysis: Variance explained in the global effectiveness of total hip arthroplasty at 12 months follow-up by sociodemographic and medical variables, preoperative expectations, change in symptoms and the fulfilment of expectations (complete case analysis).

## Data Availability

The datasets used and/or analyzed during the current study are available from the corresponding author on reasonable request.

## Abbreviations

THA: Total Hip Arthroplasty
NRS: Numeric Rating Scale
CPG: Chronic Pain Grade
MPSS: Mainz Pain Staging System
PPT: Pressure Pain Threshold
WOMAC: Western Ontario and McMaster Universities Osteoarthritis Index
SF-12: Short-Form Health Survey
DASS: Depression, Anxiety, Stress Scales
PHQ: Patient Health Questionnaire
TSK: Tampa Scale for Kinesiophobia
KPI: Kiel Pain Inventory
CTS: Catastrophizing Thought Scale
THS: Thoughts of Helplessness Scale
TSS: Thought Suppression Scale
NASS: North American Spine Society
τB: Kendall Tau-B
