# Supplementary material for: The virtue of optimistic realism - expectation fulfillment predicts patient-rated global effectiveness of total hip arthroplasty
Source: BMC Musculoskelet Disord. 2021 Feb 13;22:180. doi: 10.1186/s12891-021-04040-y (PMC7882076; doi:10.1186/s12891-021-04040-y)
Supplement: Supplementary file 1 — Additional file 1: Supplementary Figure 1. Missing value analysis of the primary outcome measure and the 14 predictor variables included sequentially into the multiple regression analysis. A. The table displays the descriptives (mean and standard deviation), the percentage of missing values per variable, the number of imputed values (missing values x number of imputations) and the type of model used for multiple imputation. For the dependent variable “global effectiveness of total hip arthroplasty” the data set was complete. More than 10% missing values were identified for the rating instrument measuring hip function and mobility (WOMAC), its symptom change score (quotient of postoperative and preoperative WOMAC score) and the calculated expectations-actuality discrepancy scores (fulfillment of expectations) of physical exercise and social interactions. B. The variable chart shows that for 93% of the predictors included at step 4 of the regression model at least 1 value is missing. The cases chart shows that 40% (N = 36) of the study participants has at least one missing value on a variable. The values chart shows that 6% of the 1260 values (cases × variables) are missing. C. The bar graph displays the percentage of study participants having none to six missing predictors. Subjects with more than three missing predictors were excluded from the analysis on the imputed data set. [file 12891_2021_4040_MOESM1_ESM.pptx]

## Slide 1
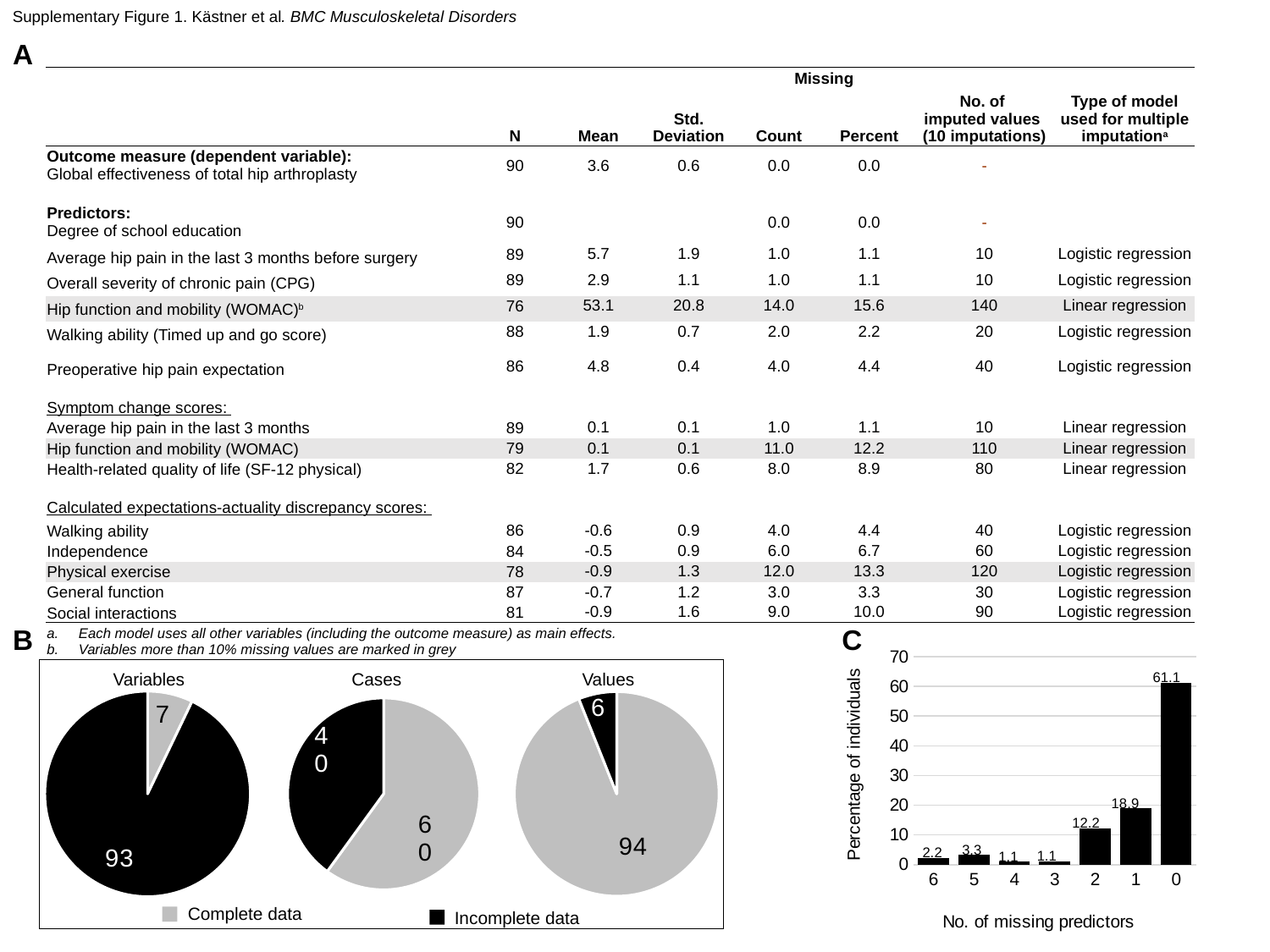

Supplementary Figure 1. Kästner et al. BMC Musculoskeletal Disorders
A
| | N | Mean | Std. Deviation | Missing | | No. of imputed values (10 imputations) | Type of model used for multiple imputationa |
| --- | --- | --- | --- | --- | --- | --- | --- |
| | | | | Count | Percent | | |
| Outcome measure (dependent variable): Global effectiveness of total hip arthroplasty | 90 | 3.6 | 0.6 | 0.0 | 0.0 | - | |
| | | | | | | | |
| Predictors: Degree of school education | 90 | | | 0.0 | 0.0 | - | |
| Average hip pain in the last 3 months before surgery | 89 | 5.7 | 1.9 | 1.0 | 1.1 | 10 | Logistic regression |
| Overall severity of chronic pain (CPG) | 89 | 2.9 | 1.1 | 1.0 | 1.1 | 10 | Logistic regression |
| Hip function and mobility (WOMAC)b | 76 | 53.1 | 20.8 | 14.0 | 15.6 | 140 | Linear regression |
| Walking ability (Timed up and go score) | 88 | 1.9 | 0.7 | 2.0 | 2.2 | 20 | Logistic regression |
| | | | | | | | |
| Preoperative hip pain expectation | 86 | 4.8 | 0.4 | 4.0 | 4.4 | 40 | Logistic regression |
| | | | | | | | |
| Symptom change scores: | | | | | | | |
| Average hip pain in the last 3 months | 89 | 0.1 | 0.1 | 1.0 | 1.1 | 10 | Linear regression |
| Hip function and mobility (WOMAC) | 79 | 0.1 | 0.1 | 11.0 | 12.2 | 110 | Linear regression |
| Health-related quality of life (SF-12 physical) | 82 | 1.7 | 0.6 | 8.0 | 8.9 | 80 | Linear regression |
| | | | | | | | |
| Calculated expectations-actuality discrepancy scores: | | | | | | | |
| Walking ability | 86 | -0.6 | 0.9 | 4.0 | 4.4 | 40 | Logistic regression |
| Independence | 84 | -0.5 | 0.9 | 6.0 | 6.7 | 60 | Logistic regression |
| Physical exercise | 78 | -0.9 | 1.3 | 12.0 | 13.3 | 120 | Logistic regression |
| General function | 87 | -0.7 | 1.2 | 3.0 | 3.3 | 30 | Logistic regression |
| Social interactions | 81 | -0.9 | 1.6 | 9.0 | 10.0 | 90 | Logistic regression |
| Each model uses all other variables (including the outcome measure) as main effects. Variables more than 10% missing values are marked in grey | | | | | | | |
B
C
### Chart
| Category | |
|---|---|
| 6 | 2.2222222222222223 |
| 5 | 3.3333333333333335 |
| 4 | 1.1111111111111112 |
| 3 | 1.1111111111111112 |
| 2 | 12.222222222222221 |
| 1 | 18.88888888888889 |
| 0 | 61.111111111111114 |
### Chart
| Category | |
|---|---|
| Complete data | 7.14 |
| Incomplete data | 92.86 |
### Chart
| Category | Cases |
|---|---|
| Complete data | 60.0 |
| Incomplete data | 40.0 |
### Chart
| Category | |
|---|---|
Variables
Cases
Values
61.1
18.9
12.2
3.3
2.2
1.1
1.1
Complete data
Incomplete data
