# Supplementary material for: The virtue of optimistic realism - expectation fulfillment predicts patient-rated global effectiveness of total hip arthroplasty
Source: BMC Musculoskelet Disord. 2021 Feb 13;22:180. doi: 10.1186/s12891-021-04040-y (PMC7882076; doi:10.1186/s12891-021-04040-y)
Supplement: Supplementary file 2 — Additional file 2: Supplementary Table 1. Correlations between baseline variables and patient-rated global effectiveness of THA. [file 12891_2021_4040_MOESM2_ESM.docx]

| **Supplementary Table 1. Correlations between baseline variables and patient-rated global effectiveness of THA** | |  |
| --- | --- | --- |
| **Baseline and psychological variables** | **Global effectiveness of THA** |  |
| **Sociodemographic variables and Body Mass Index** | **Correlation coefficient (p-value)^b^** |  |
| Gender | phi=0.255 (p=0.118) |  |
| Age at examination, years | Pearson’s r=-0.47 (p=0.658) |  |
| Degree of school education | Kendall Τau_B_ (T_B_)=0.301 (**p=0.002**) |  |
| Body Mass Index, BMI*,* kg/m^2^ | Pearson’s r=-0.247 (*p=0.019*) |  |
| **Pain characteristics** | **Correlation coefficient (p-value)^c^** |  |
| Average hip pain in the last 3 months before surgery, Numeric Rating Scale (0-10) | T_B_=-0.330 (**p<0.001**) |  |
| Overall severity of chronic pain condition, CPG (von Korff) | T_B_=-0.375 (**p<0.001**) |  |
| Pain chronicity, MPSS^d^ | T_B_=-0.229 (*p=0.021*) |  |
| Duration of hip pain | T_B_=0.109 (p=0.256) |  |
| Pressure pain threshold (PPT) | r=0.190 (p=0.075) |  |
| **Functional capacity** | **Kendall Τau_B_ (p-value)^e^** |  |
| Walking ability, Timed up and go test score | T_B_=-0.313 (**p=0.002**) |  |
| Hip function and mobility, WOMAC^f^ | T_B_=-0.311 (**p=0.001**) |  |
| **Psychological variables** | **Kendall Τau_B_ (p-value)^g^** |  |
| Health-related quality of life, SF-12^h^ |  |  |
| SF-12 Physical | T_B_=-0.020 (p=0.825) |  |
| SF-12 Mental | T_B_=0.052 (p=0.562) |  |
| Psychological distress, DASS^i^ |  | |
| DASS Depression | T_B_=-0.134 (p=0.143) |  |
| DASS Anxiety | T_B_=-0.085 (p=0.373) |  |
| DASS Stress | T_B_=-0.128 (p=0.157) |  |
|  |  |  |
| Somatization, PHQ-15^j^ | T_B_=-0.011 (p=0.911) |  |
| Kinesiophobia, TSK^k^ | T_B_=-0.074 (p=0.433) |  |
| Cognitive appraisal of pain*,* KPI^l^ |  |  |
| Catastrophizing thought scale | T_B_=-0.051 (p=0.602) |  |
| Helplessness scale | T_B_=-0.218 (*p=0.019*) |  |
| Thought suppression scale | T_B_=-0.230 (*p=0.014*) |  |
|  |  |  |
| Fear of surgery | T_B_=-0.187 (*p=0.040*) |  |
| Fear of pain after the surgery | T_B_=-0.089 (p=0.341) |  |
| *Multiple testing adjusted significances (applying Bonferroni to each variable cluster) are set in boldface. Nominally significant p-values are set in italics. Variables significantly associated with global effectiveness of THA were included as step 1 in the hierarchical multiple regression analysis (Table 3).*  *^a^Correlation analyses are based on N=76-90 subjects due to varying numbers of missing data per variable; ^b^Bonferroni-adjusted significance level: p=0.05/4; ^c^Bonferroni-adjusted significance level: p=0.05/3 ^d^MPSS=Mainz Pain Staging System (Gerbershagen et al., 2008); ^e^Bonferroni-adjusted significance level: p=0.05/2; ^f^WOMAC=Western Ontario and McMaster Universities Osteoarthritis Index (Stucki et al., 1996); ^g^Bonferroni-adjusted significance level: p=0.05/12; ^h^SF-12=short form of the Health Survey Questionnaire (Jenkinson et al., 1997); ^i^DASS=Depression, Anxiety, Stress Scales (Nilges und Essau, 2015); ^j^PHQ-15=Patient Health Questionnaire (Kroenke et al. 2002); ^k^TSK=* *Tampa Scale for Kinesiophobia (Roelofs et al. 2004); ^l^KPI=Kiel Pain Inventory (Hasenbring, 1994)* | |  |
