# Supplementary material for: The virtue of optimistic realism - expectation fulfillment predicts patient-rated global effectiveness of total hip arthroplasty
Source: BMC Musculoskelet Disord. 2021 Feb 13;22:180. doi: 10.1186/s12891-021-04040-y (PMC7882076; doi:10.1186/s12891-021-04040-y)
Supplement: Supplementary file 3 — Additional file 3: Supplementary Table 2. Correlations between preoperative expectations, change in symptoms and calculated expectations-actuality discrepancy scores with the patient-rated global effectiveness of THA. [file 12891_2021_4040_MOESM3_ESM.docx]

| **Supplementary Table 2. Correlations between preoperative expectations, change in symptoms and calculated expectations-actuality discrepancy scores with the patient-rated global effectiveness of THA** | |
| --- | --- |
| **Pre-operative expectations^a^** | **Global effectiveness of THA**  **Kendall Τau_B_ (p-value)^b^** |
| Hip pain | T_B_=0.329 *(***p=0.002***)* |
| Back pain | T_B_=-0.101 (p=0.325) |
| Walking ability | T_B_=0.163 (p=0.122) |
| Independence | T_B_=0.064 (p=0.533) |
| Physical exercise | T_B_=0.095 (p=0.355) |
| General function | T_B_=-0.045 (p=0.664) |
| Social interactions | T_B_=-0.196 (*p=0.047*) |
| Mental well-being | T_B_=-0.006 (p=0.949) |
| **Change in symptoms^c^** | **Global effectiveness of THA**  **Kendall Τau_B_ (p-value)^d^** |
| Average hip pain in the last 3 months | T_B_=-0.322 (**p=0.002**) |
| Overall severity of chronic pain condition, CPG (von Korff) | T_B_=-0.146 (p=0.122) |
| Hip function and mobility, WOMAC | T_B_=-0.295 (**p=0.002**) |
| Health-related quality of life, SF-12^h^ |  |
| SF-12 Physical | T_B_=0.304 (**p=0.001**) |
| SF-12 Mental | T_B_=0.011 (p=0.899) |
| Psychological distress, DASS^i^ |  |
| DASS Depression | T_B_=-0.211 (*p=0.049*) |
| DASS Anxiety | T_B_=0.064 (p=0.600) |
| DASS Stress | T_B_=-0.053 (p=0.586) |
| Kinesiophobia, TSK^k^ | T_B_=-0.243 (*p=0.011*) |
| **Calculated expectations-actuality discrepancy scores^e^** | **Global effectiveness of THA**  **Kendall Τau_B_ (p-value)^b^** |
| Hip pain | T_B_= 0.157 (p=0.126) |
| Back pain | T_B_=0.101 (p=0.346) |
| Walking ability | T_B_=0.473 (**p<0.0001**) |
| Independence | T_B_=0.471 (**p<0.0001**) |
| Physical exercise | T_B_=0.340 (**p=0.001**) |
| General function | T_B_=0.314 (**p=0.001**) |
| Social interactions | T_B_=0.317(**p=0.001**) |
| Mental well-being | T_B_=0.215 (*p=0.026*) |
| *Multiple testing adjusted significances (applying Bonferroni to each variable set) are set in boldface. Nominally significant p-values are set in italics. Variables significantly associated with global effectiveness of THA were included as steps 2 to 4 in the hierarchical multiple regression analysis (Table 3).*  *^a^Correlation analyses are based on N=79-90 subjects due to varying numbers of missing data per variable; ^b^Bonferroni-adjusted significance level: p=0.05/8; ^c^Correlation analyses are based on N=54-90 subjects due to varying numbers of missing data per variable; change scores were obtained by dividing the outcome measures at 12 months follow-up by the preoperative scores; ^d^Bonferroni-adjusted significance level: p=0.05/9;  ^c^Correlation analyses are based on N=69-90 subjects due to varying numbers of missing data per variable* | |
