# Supplementary material for: The virtue of optimistic realism - expectation fulfillment predicts patient-rated global effectiveness of total hip arthroplasty
Source: BMC Musculoskelet Disord. 2021 Feb 13;22:180. doi: 10.1186/s12891-021-04040-y (PMC7882076; doi:10.1186/s12891-021-04040-y)
Supplement: Supplementary file 4 — Additional file 4: Supplementary Table 3. Results of the sequential multiple regression analysis: Variance explained in the global effectiveness of total hip arthroplasty at 12 months follow-up by sociodemographic and medical variables, preoperative expectations, change in symptoms and the fulfilment of expectations (complete case analysis). [file 12891_2021_4040_MOESM4_ESM.docx]

| **Supplementary Table 3. Results of the sequential multiple regression analysis: Variance explained in the global effectiveness of total hip arthroplasty at 12 months follow-up by sociodemographic and medical variables, preoperative expectations, change in symptoms and the fulfilment of expectations (complete case analysis)** | | | | |
| --- | --- | --- | --- | --- |
|  | **Variables included  upon each step** | **R^2^** | **R^2^_change_ (p-value)** | **β (p-value) in final model (only significant  predictor variables shown)** |
| **Step 1** (N=72): Confounding factors | Degree of school education  Average hip pain in the last 3 months before surgery  Overall severity of chronic pain (CPG)  Hip function and mobility (WOMAC)  Walking ability (Timed up and go score) | 0.332 | - | β= -0.363 (p=0.002)  Timed up and go score |
| **Step 2** (N=68): Preoperative expectations | Degree of school education  Average hip pain in the last 3 months before surgery  Overall severity of chronic pain (CPG)  Hip function and mobility (WOMAC)  Walking ability (Timed up and go score)  **Preoperative hip pain expectation** | 0.417 | 0.085 (p<0.001) | β= -0.363 (p=0.002)  Timed up and go score  β= 0.276 (p=0.006)  Hip pain expectation |
| **Step 3** (N=62): Improvement in symptoms (Symptom change scores) | Degree of school education  Average hip pain in the last 3 months before surgery  Overall severity of chronic pain (CPG)  Hip function and mobility (WOMAC)  Walking ability (Timed up and go score)  Preoperative hip pain expectation  **Symptom change scores:**  **Average hip pain in the last 3 months**  **hip function and mobility (WOMAC)**  **Health-related quality of life (SF-12 physical)** | 0.417 | 0.056 (p<0.0001) | β= -0.308 (p=0.010)  Timed up and go score  β= 0.327 (p=0.003)  Hip pain expectation |
| **Step 4** (N=54): Fulfillment of expectations (calculated expectations-actuality discrepancy scores) | Degree of school education  Average hip pain in the last 3 months before surgery  Overall severity of chronic pain (CPG)  Hip function and mobility (WOMAC)  Walking ability (Timed up and go score)  Preoperative hip pain expectation  Symptom change scores:  Average hip pain in the last 3 months  Hip function and mobility (WOMAC)  Health-related quality of life (SF-12 physical)  **Calculated expectations-actuality discrepancy scores:**  **Walking ability**  **Independence**  **Physical exercise**  **General function**  **Social interactions** | 0.733 | 0.260 (p<0.0001) | β= 0.504 (p<0.0001)  Hip pain expectation  β= 0.407 (p=0.004)  Fulfillment of expectations (calculated expectations-actuality discrepancy scores):  *Walking ability*  β= 0.407 (p=0.025)  Fulfillment of expectations (calculated expectations-actuality discrepancy scores):: *Independence* |
| **Final model** (N=82; only significant predictors included) | Preoperative hip pain expectation  Calculated expectations-actuality discrepancy scores:  Walking ability  Independence | 0.402 |  |  |
| *The variables marked in bold are added up to the model at each step; due to varying numbers of missing data per variable, sample sizes vary. Predictor variables individually significantly associated with global effectiveness of THA (see Supplementary Table 1 and 2) were entered in four steps. The significant predictors in the final model were: Hip pain expectation and the calculated expectations-actuality discrepancy scores of walking ability and independence in everyday life.*  *β in final model= β regression coefficient after all listed variables have been entered; R2change= Increase in explained variance by step; adjusted R2, = R2-(k-1)/ (n-k)*(1- R2) where n=no. observations, k=no. independent variables. Level of significance was set to p<0.05.* | | | | |
